# Antitumor and antiangiogenic activity of the novel chimeric inhibitor animacroxam in testicular germ cell cancer

**DOI:** 10.1002/1878-0261.12582

**Published:** 2019-10-22

**Authors:** Gustav Steinemann, Alexandra Dittmer, Jacob Schmidt, David Josuttis, Michael Fähling, Bernhard Biersack, Nicola Beindorff, Eva Jolante Koziolek, Rainer Schobert, Winfried Brenner, Thomas Müller, Bianca Nitzsche, Michael Höpfner

**Affiliations:** ^1^ Corporate Member of Freie Universität Berlin Berlin Institute of Health Institute of Physiology Humboldt‐Universität zu Berlin Charité – Universitätsmedizin Berlin Germany; ^2^ Corporate Member of Freie Universität Berlin Berlin Institute of Health Institute of Vegetative Physiology Humboldt‐Universität zu Berlin Charité – Universitätsmedizin Berlin Germany; ^3^ Department of Organic Chemistry University of Bayreuth Germany; ^4^ Corporate Member of Freie Universität Berlin Berlin Institute of Health Berlin Experimental Radionuclide Imaging Center (BERIC) Humboldt‐Universität zu Berlin Charité – Universitätsmedizin Berlin Germany; ^5^ German Cancer Research Center (DKFZ) Heidelberg Germany; ^6^ German Cancer Consortium (DKTK) Berlin Germany; ^7^ Clinic of Internal Medicine IV ‐ Hematology and Oncology Division Universitätsklinikum Halle (Saale) Germany

**Keywords:** cancer therapy, chick chorioallantoic membrane, HDAC inhibitors, PET/MR imaging, tumor angiogenesis

## Abstract

Chimeric inhibitors, which merge two drug pharmacophores in a single molecule have become a prominent approach for the design of novel anticancer compounds. Here, we examined animacroxam, which combines histone deacetylase (HDAC) inhibitory and cytoskeleton‐interfering pharmacophores, in testicular germ cell tumors (TGCT). The effectiveness of animacroxam was compared to that of the commonly applied chemotherapeutic cisplatin as well as the clinically approved HDAC inhibitor vorinostat. The antineoplastic and antiangiogenic effects of animacroxam on TGCT *in vivo* were assessed through exploratory animal studies and a modified chorioallantoic membrane assay, revealing that animacroxam has significant antitumor activity in TGCT. A novel positron emission tomography/MR‐imaging approach was applied to determine tumor volume and glucose [2‐fluoro‐2‐deoxy‐d‐glucose (18F‐FDG)] uptake in TGCT tumors, revealing reduced glucose uptake in animacroxam‐treated TGCTs and showing a dose‐dependent suppression of glycolytic enzymes, which led to a breakdown in glycolytic energy production. Furthermore, the observed antiangiogenic effects of animacroxam were related to its ability to inhibit endothelial cell–cell communication, as the expression of gap junction‐forming connexin 43 was strongly suppressed, and gap‐junctional intercellular mass transport was reduced. Our data suggest that the chimeric HDAC inhibitor animacroxam may become a promising candidate for the treatment of solid cancers and may serve as an interesting alternative to platinum‐based therapies.

## Graphical abstract

The chimeric inhibitor animacroxam combining histone deacetylase (HDAC)‐inhibiting and cytoskeleton‐interfering pharmacophores exerts pronounced antineoplastic efficiency. Animacroxam‐inhibited tumor growth is associated with a breakdown of tumor cell glucose metabolism and antiangiogenic effects involving the disruption of endothelial cell–cell communication. As the effectiveness of the novel chimeric inhibitor goes beyond that of HDAC inhibition alone, animacroxam may become an interesting compound for future cancer therapy.

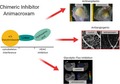

**Abbreviations**
18F‐FDG: 2‐fluoro‐2‐deoxy‐d‐glucose
BPGM: bisphosphoglycerate mutase
CAM: chorioallantoic membrane
Cbx: carbenoxolone
Cx43: connexin 43
GJIC: gap‐junctional intercellular communication
HDAC: histone deacetylase
HDACi: histone deacetylase inhibitor
HK: hexokinase
i.v.: intravenous
mRNA: messenger ribonucleic acid
MRT: magnet resonance tomography
NaCl: sodium chloride
PET: positron emission tomography
ROS: reactive oxygen species
RT‐PCR: reverse transcription polymerase chain reaction
SUV: standardized uptake value
TGCT: testicular germ cell tumor
VOI: volume of interest

## 1 Introduction

Molecular hybridization has emerged as a smart and effective approach in the development of novel anticancer compounds. Two drug pharmacophores are merged to one single molecule, which acts on two distinct cellular targets. This contrasts with combination therapies, where two or more active compounds are given sequentially or as a cocktail to achieve (over‐)additive antitumoral effects. Combination therapies often cause problems, such as different drug solubilities or physical incompatibilities that can result in precipitation or drug inactivation. Moreover, the risk of drug–drug interactions and the occurrence of adverse or unwanted side effects require a complex dose adjustment to avoid therapeutic ineffectiveness. To overcome these limitations, several research groups started to develop so‐called chimeric compounds. Especially, chimeric agents linking histone deacetylase (HDAC) inhibitory pharmacophores with protein kinase inhibitors, DNA‐damaging compounds or cytoskeleton targeting pharmacophores have recently gained much attention (Hesham *et al.*, 2018; Schobert and Biersack, 2017). HDACs play a pivotal role in cellular chromatin remodeling and are implicated in the epigenetic regulation of cell metabolism, growth, and differentiation (Allfrey *et al.*, 2016; Hongs *et al.*, 1993; Jones *et al.*, 2000). In cancer, HDACs are often overexpressed or overactive and repress the expression of tumor suppressor genes leading to uncontrolled proliferation and insensitivity to cell repair mechanisms and apoptosis. Thus, HDAC inhibitors (HDACi) have become promising compounds for innovative therapeutic approaches of several cancers (Jones *et al.*, 2016). However, the effectiveness of HDACi is often hampered by acquired drug resistance and associated enhanced tumor aggressiveness. Hence, hybrid compounds combining HDAC‐inhibiting moieties with other cancer targets have been developed to overcome drug resistance and to synergistically enhance the anticancer activity of these novel compounds (Schobert and Biersack, 2017).

Recently, we introduced the novel hybrid compound animacroxam combining HDAC‐inhibitory hydroxamic acid with a cytoskeleton‐interfering 4,5‐diarylimidazole moiety as a highly effective chimeric compound with pronounced antiproliferative, apoptotic, cell cycle arresting and antimigratory effects (Steinemann *et al.*, 2017). Animacroxam did not cause unspecific cytotoxicity and, most notably, was shown to exert its effects irrespective of the cisplatin sensitivity of the investigated cancer cell models. Cisplatin is one of the most effective chemotherapeutic agents for several human cancers including lung, ovarian, cervix, breast, and germ cell cancer (Cohen and Lippard, 2001; Dasari and Tchounwou, 2015; Florea and Büsselberg, 2011). However, it is also known to be highly toxic causing nephro‐, hepato‐, and cardiotoxicities (Miller *et al.*, 2010; Patanè, 2014), making cisplatin not applicable for several subgroups of patients (elderly patients, impaired kidney function, or comorbidities) (Dasari and Tchounwou, 2015).

If animacroxam proofs to exert antineoplastic effectiveness comparable to that of cisplatin, it might be a promising alternative for the treatment of cisplatin‐refractory tumors and for patients that are not applicable for platinum‐based therapies (Dasari and Tchounwou, 2015). Therefore, we here investigated the antineoplastic and antiangiogenic effectiveness of animacroxam *in vivo* and compared it to that of cisplatin. The underlying modes of action of animacroxam were further deciphered in terms of tumor cell energy metabolism and gap‐junctional communication of tumor angiogenic endothelial cells. To compare the potencies of the HDACi, the effects of animacroxam were contrasted with those of the clinically relevant HDACi vorinostat.

For the *in vivo* evaluations, xenografted mice and an advanced chorioallantoic membrane (CAM) assay model were employed. The CAM is a highly vascularized membrane of fertilized chicken eggs, which serves as an embryo‐feeding microvascular network for the supply with oxygen and nutrients. The immune‐incompetent CAM can be easily inoculated with human tumors or cell culture material. However, in CAM assays a precise tumor volumetric analysis is difficult to define and therefore conventional determinations via microscopic analysis or tumor weighing at the end of the experiment come with considerable deviations (Ribatti, 2014). Furthermore, treatment‐induced metabolic changes of the tumors can only be estimated by immunohistochemical staining and changes of an individual tumor over time are impossible. To overcome these limitations, we developed an advanced CAM assay by employing state‐of‐the‐art magnetic resonance imaging (MRI)/positron emission tomography (PET) to precisely calculate tumor volume and to perform metabolic assessments of individual tumors in a noninvasive manner (Ma *et al.*, 2015; Warnock *et al.*, 2013).

## 2 Materials and methods

### 2.1 Compounds

Stock solutions of animacroxam (20 mm, synthesized according to procedure (Mahal, Schruefer, *et al.*, 2015)), vorinostat (20 mm; Sigma‐Aldrich, St. Louis, Mo, USA), and carbenoxolone (Cbx) (50 mm; Sigma) were prepared in DMSO and stored at 4 °C. Cisplatin (TEVA, Ulm, Germany) was used as a concentrate of 1 mg·mL^−1^ in 0.9% NaCl. In all experiments, the final DMSO concentration was < 0.25%. The concentrations of animacroxam and vorinostat used in the experiments were deduced from previous studies, in which the half‐maximal inhibitory concentration values of TGCT and endothelial cells were determined (Mahal, Schruefer, *et al.*, 2015; Steinemann *et al.*, 2017). The concentrations used here for *in vitro* studies refer to the previously determined concentration ranges of animacroxam of 0.5–2.5 µm for 2102EP and endothelial EA.hy926 cells.

### 2.2 Cell culture

2102EP testicular germ cell cancer cells (nonseminoma, teratocarcinoma, and yolk‐sack tumor), kindly provided by F. Honecker (St. Gallen, Switzerland), and somatic hybrid endothelial EA.hy926 cells (American Type Culture Collection® CRL‐2922™) were cultured in Dulbecco’s modified Eagle’s medium/F12 (1 : 1) medium supplemented with 10% FBS, 2.0 mm l‐glutamine, 50 U·mL^−1^ penicillin, and 50 µg·mL^−1^ streptomycin (all from Life Technologies, Carlsbad, CA, USA) and maintained in an incubator (5% CO_2_, 37 °C, humidified atmosphere).

### 2.3 Mice studies

The investigation of this study was approved by the Laboratory Animal Care Committee of Sachsen‐Anhalt, Germany. To generate xenograft tumors, 8.0 × 10^6^ 2102EP cells were resuspended in PBS and injected subcutaneously into the flank of 8‐week‐old athymic nude mice (*n* = 4) (Charles River, Göttingen, Germany). Treatment was started when the tumors had reached a volume of at least 150 mm^3^. Mice were divided into two groups with similar mean tumor volumes. Animacroxam was resuspended in Tween‐80/EtOH/saline at a concentration of 6 mg·mL^−1^. Mice received a daily dose of 60 mg·kg^−1^ body weight by oral gavage (0.01 mL·g^−1^) on four consecutive days. The control group received vehicle. The tumor volumes were calculated by caliper measurement using the formula *a* ^2^ × *b* × 0.5 with *a* being the short and *b* the long dimension. Body weight and behavior of mice were analyzed daily during treatment.

### 2.4 Chorioallantoic membrane (CAM) assay

Fertilized specific pathogen‐free chicken eggs (Gallus gallus; VALO Biomedia, Cuxhaven, Germany) were maintained and handled as described earlier (Mahal, Schruefer, *et al.*, 2015).

#### 2.4.1 CAM xenografts

For the preparation of tumor plaques, 10 × 10^6^ 2102EP cells were mixed with 150 µL matrigel (Corning Life Sciences, Tewksbury MA, USA). The cell suspension was pipetted into a 6‐well plate and stored for at least 4 h in the incubator (5% CO_2_, 37 °C, humidified atmosphere) to form stable plaques. Thereafter, medium was added to each well and the plaques were maintained in the incubator overnight. The tumor plaques were implanted to the CAM of 7‐day‐old chicken eggs and maintained for 3 days to allow angiogenic connection of the tumors to the CAM. For treatment, CAMs were injected intravenously at day ten of chicken embryonic development with animacroxam (5 µm), cisplatin (2.5 µm), or NaCl (0.9%), using a 30G syringe attached to a catheter prior to first MRI scan (Day 0). Final concentrations were calculated by assuming a total blood volume of 1.0 mL (Barnes and Jensen, 1959; Kind, 1975). The chicken eggs were then maintained for 1 week in an incubator without further drug injection. Thereafter, a second MRI scan was performed at the end of the experiment (Day 7) and the treatment‐induced changes in tumor volume were determined by calculation and comparison of the initial tumor volume at day 0 with that of day 7.

#### 2.4.2 In vivo magnetic resonance imaging (MRI)/positron emission tomography (PET)

Tumor volume was determined by MR imaging prior to and after 7 days of treatment using a dedicated small animal 1 Tesla nanoScan PET/MRI (Mediso, Budapest, Hungary). To avoid motion artifacts of the embryo during MRI scans, eggs were cooled down for 1 h at 4 °C before MRI scanning started at room temperature. Anatomical MRI scans were acquired using a T1‐weighted 3D spoiled gradient echo sequence (T1 GRE 3D) with the following parameters: coronal as well as transverse orientation, matrix 224 × 224 × 22, with a voxel size of 0.29 × 0.29 × 0.5 mm^3^, TR: 50 ms, TE: 2.7 ms, and flip angle 40° and a T2‐weighted 2D turbo‐spin echo sequence with the following parameters: matrix 252 × 252 × 22, with a voxel size of 0.29 × 0.29 × 0.7 mm^3^, TR: 8885 ms, TE: 100 ms, and four number of averages. MR images were analyzed using the interviewFUSION software (Mediso, version 3.01.004.000). To determine total tumor volume, a volume of interest (VOI) was manually contoured, based on the T1w and T2w MR images.

Positron emission tomography imaging of tumor glucose metabolism was performed by injecting 0.1 mL of 12 mega‐Becquerel ^18^F‐fluoro‐deoxyglucose (18F‐FDG) into a CAM vein. PET was performed at 37 °C starting 40 min after injection of the tracer. The uptake of 18F‐FDG in the tumor tissue was determined by manual contouring of a VOI of the PET image using PMOD 3.5 (PMOD Technologies Ltd., Zürich, Switzerland). An average standardized uptake value (SUV) was computed from the 10 hottest voxels (SUVmax10).

#### 2.4.3 Immunohistochemistry

At the end of the experiments, the tumors were excised from the CAM and fixed in 4% formalin. Paraffin‐embedded tissue was cut into sections (1–2 µm) representing the rim and the middle of the tumor. For immunohistochemistry, sections were dewaxed and subjected to a heat‐induced epitope retrieval step prior to incubation with either anti‐Ki67 (clone MIB‐1; Agilent Technologies, Santa Clara, CA, USA) or anti‐desmin (clone D33; Agilent Technologies). Cytokeratin epitope retrieval was performed protein‐induced, followed by incubation with anti‐cytokeratin (Lu‐5; Merck Millipore, Darmstadt, Germany). Streptavidin–biotin labeling employing Dako REAL™ Detection System, alkaline phosphatase/RED, and EnVision Detection System, Peroxidase/DAB, Mouse (Agilent Technologies) was used for visualization. Nuclei were counterstained with hematoxylin (Merck Millipore). Negative controls were performed by omitting the primary antibody. The quantification of the Ki67‐ and desmin‐stained tumor sections was carried out on three slices from the rim and three slices from the middle of each control and treated tumor. For calculation, 10 high‐power fields (HPF, 0.237 mm^2^) of each rim and middle section were selected randomly in a blinded manner and the number of Ki67‐ or desmin‐positive cells within the HPFs was counted. Then, the relative number of Ki67‐ or desmin‐expressing cells compared to the control‐treated tumors was calculated. Images were acquired using the Axio Imager Z1 microscope (Zeiss, Wetzlar, Germany). All evaluations were performed in a blinded manner.

#### 2.4.4 Blood vessel analysis

At day 10–12 of the embryonic development, a silicone ring (Ø 5 mm) was placed on the CAM for topical treatment with animacroxam, vorinostat, or PBS (control). Images were taken after 48 h using a stereomicroscope equipped with a Di‐Li 1009‐SHD HD‐Digital Camera System (Distelkamp‐Electronic, Kaiserslautern, Germany). Images were macroscopically examined by counting the number of blood vessels of treated and untreated areas of the same CAM.

Capillary perfusion and microcirculation were assessed by intravital video‐microscopy. Video sequences (10 s each) of treated and untreated CAM areas were recorded (CMOS camera; Sony ICE 6000, Tokyo, Japan) and processed as described earlier (Xiang *et al.*, 2017) allowing to generate a single image visualizing the lumen and blood perfusion of small capillaries in the vascular network.

#### 2.4.5 Confocal immunofluorescence microscopy

Chorioallantoic membrane sections were excised, and after washing (PBS), fixation (4% formalin), permeabilization (0.2% Triton X/PBS), and blocking (1% goat serum, 2% BSA, 0.1% sodium acid, and 0.2% Triton X solution), primary anti‐desmin (clone D33; Agilent Technologies) was incubated overnight at 4 °C. After incubation with secondary fluorescence‐coupled antibodies (goat anti‐mouse 568, A11031; Invitrogen, Waltham, MA, USA) for 1 h at RT, CAMs were embedded in ProLong Gold antifade reagent (Thermo Fisher, Waltham, MA, USA) and analyzed using a confocal microscope (Leica Sp5, Wetzlar, Germany) equipped with 20/40/63× objectives.

### 2.5 Hexokinase activity

Fluorometric determination of hexokinase (HK) activity was performed according to the manufacturer’s protocol (Abcam, Cambridge, MA, USA). 2102EP cells were seeded in a 6‐well plate at 80% of confluence. Changes in HK activity were calculated after 24 or 48 h of treatment, and the results are given as the percentage of HK activity of treated samples compared to HK activity of untreated cells, which was set 100%.

### 2.6 Quantification of lactate levels

Anaerobic glycolysis was determined by measuring lactate levels in the supernatant of treated TGCT cells using an ABL825 Flex Blood‐gas analyzer (Radiometer GmbH, Krefeld, Germany). Values were normalized to the corresponding total protein level per sample, as an estimate of cell number.

### 2.7 Measurement of reactive oxygen species (ROS)

Formation of cytosolic ROS after treatment with animacroxam, vorinostat, or brimamin was determined with the ROS‐sensitive fluorescent dye CellROX Orange (Schmidt *et al.*, 2018) (Thermo Fisher). H_2_O_2_ served as a positive control. Fluorescence images (ex/em 546/575 nm) of dye‐loaded cells (1.0 µm) were taken on an Axioskop 40 microscope (Zeiss) equipped with a digital camera system (DX4‐285FW; Kappa Optronics, Gleichen, Germany). GSH measurement (Starheim *et al.*, 2016) was carried out using the GSH‐GLO Glutathione Assay (Promega, Mannheim, Germany) according to the manufacturer`s protocol.

### 2.8 Tube formation assay

2.0 × 10^4^ EA.hy926 cells per well were cultured in matrigel‐coated (Corning Life Sciences) cell culture plates (Ibidi, Munich, Germany). After 16 h of treatment with animacroxam or vorinostat, pictures were taken with an Eclipse T microscope (Nikon Europe, Amsterdam, Netherlands). Quantification of tube formation was performed manually by counting the number of capillary‐like tubes.

### 2.9 Scrape loading assay

Confluent layers of EA.hy926 cells grown on glass coverslips were treated for 24 h. Coverslips were mounted in a scrape chamber, and a fine cut line was scraped into the monolayer using a razor blade. Scraped coverslips were incubated with Lucifer Yellow (Sigma), washed with PBS fixed in 4% paraformaldehyde. Fluorescence images of the distribution pattern of Lucifer Yellow were taken with an Eclipse T fluorescence microscope (Nikon). Eight fluorescence images per scrape were obtained. Images of the unscratched monolayer served for background determination. imagej software (version 2.0.0, LOCI, University of Wisconsin, Madison, WI, USA) was used to calculate dye diffusion width as described earlier (Bader *et al.*, 2017). Means ± SEM of *n* = 4–16 measurements per condition were calculated and normalized to vehicle‐treated controls.

### 2.10 Western blot

Western blotting was performed as described before (Höpfner *et al.*, 2004). The following antibodies were used: anti‐Cx43 (Sigma), anti‐tubulin (Thermo Fisher), anti‐GAPDH (Calbiochem; Merck), anti‐bisphosphoglycerate mutase (BPGM; Novus Biologicals, Littleton CO, USA), anti‐tubulin ß‐2B, (OriGene, Rockville MD, USA), anti‐GLUT1 (Thermo Fisher), and secondary horseradish‐peroxidase‐coupled antibodies (Vector Laboratories, Burlingame, CA, USA). Membranes were developed using Amersham ECL Prime Western Blotting Detection Reagent (GE Healthcare Life Sciences, Freiburg, Germany) and a Fusion SL camera (Vilber Lourmat, Eberhardzell, Germany). For quantification, imagej was used and the density of the protein bands was either normalized to tubulin (GLUT‐1, BPGM) or GAPDH (Cx43) loading control.

### 2.11 Statistical analysis

Statistical analyses were performed with graphpad (version 7.00). Except stated otherwise, all experiments were independently repeated *n* = 3–5 times and the results are presented as means ± SEM. Statistical significance was tested with (un)paired *t*‐test or one‐way ANOVA with *post hoc* Tukey's multiple comparison test using graphpad prism 8.0.0 (GraphPad Software, San Diego, CA, USA).

## 3 Results

### 3.1 Antineoplastic effects of animacroxam

**Figure 1:**
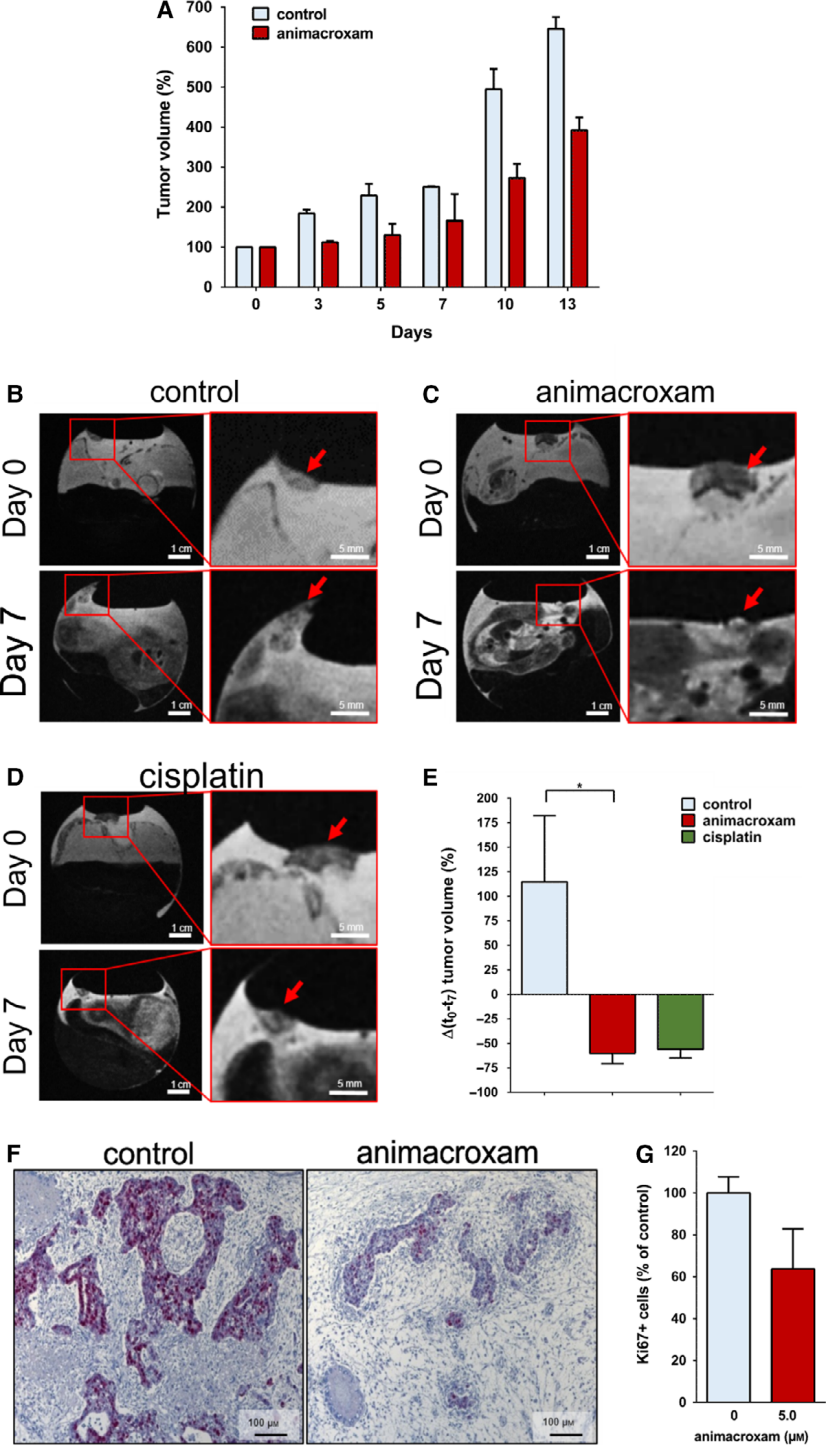
Antineoplastic effects of animacroxam and cisplatin *in vivo*. (A) Relative tumor volume of TGCT xenografts grown in athymic nude mice after the treatment with 0.9% NaCl or animacroxam (daily dose of 60 mg·kg^−1^ body weight on four consecutive days, intraperitoneal). Results are shown as mean ± SEM of *n* = 2 mice in each group. (B–D) T2w MR images of tumor‐bearing CAMs of fertilized chicken eggs taken before and after 7 days of a single intravenous injection with 0.9% NaCl (B), 5.0 µm animacroxam (C), or 2.5 µm cisplatin (D); scale bar = 1 cm (left side) or 0.5 cm (right side). (E) Mean changes in tumor volume of *n* = 3 CAM experiments. (F) IHC staining of Ki67 (red–violet) revealed a pronounced reduction of proliferation in animacroxam‐treated tumors as compared to untreated controls; scale bar = 100 µm. (G) Quantification of Ki67‐positive cells as a marker for proliferation in tumors excised out of the CAM after incubation with animacroxam (5.0 µm). Results are shown as mean ± SEM of *n* = 4 independent preparations. *P‐values of ≤ 0.05, unpaired *t*‐test.

Testicular germ cell tumors‐bearing athymic nude mice were treated with the chimeric imidazole‐derivative animacroxam to determine its antineoplastic efficiency *in vivo* for the first time. While the relative tumor volume of vehicle‐treated control mice increased within 14 days, animacroxam (60 mg·kg^−1^)‐treated tumors showed a reduced relative growth as compared to control tumors (Fig. 1A). Additionally, animacroxam exerted a good biotolerability as no changes in behavior, weight, or food and water consumption of the mice were observed. This confirmed prior toxicity studies in which we already showed an excellent tolerability of imidazole‐based chimeric inhibitors in mice treated with of up to > 150 mg·kg^−1^ body weight/day (Höpfner *et al.*, 2018).

The encouraging findings of the initial mouse experiments prompted us to investigate the antineoplastic potency and underlying modes of action of animacroxam in more depth by employing a modified and advanced CAM assay. A novel multiparametric *in vivo* imaging with MRI/PET allowed us to precisely monitor the individual tumor development of TGCT microtumors inoculated onto the blood vessel network of the CAM in a noninvasive manner (Fig. 1). Three days after inoculation, the microtumors got attached and connected to the CAM and were then treated with a single intravenous injection of either animacroxam, cisplatin, or NaCl (vehicle treatment). In prior dose‐finding experiments, we determined the most effective but still well‐tolerated drug concentration of animacroxam and cisplatin for intravenous injection. Here, animacroxam concentrations of 5.0–7.5 µm were highly effective without affecting the development and survival of the chicken embryos. Thus, for animacroxam 5.0 µm was chosen as a safe and effective concentration. In case of cisplatin, doses of 2.0 and 2.5 µm were shown to be most effective and still well tolerated, while cisplatin concentrations of 3.0 µm or above resulted in a significant delay in embryo development and an increase in the number of embryo death during the treatment. Thus, we decided to apply 2.5 µm of cisplatin as an effective and safe concentration.

The size of vehicle‐treated tumors increased by 114% within 7 days (Fig. 1B,E). Treatment with animacroxam not only stopped TGCT tumor growth but even induced a more than 60% shrinkage below the initial tumor volume at the beginning of the treatment. This reduction in tumor volume by animacroxam was statistically significant when compared to the volume of control tumors after 7 days (Fig. 1C,E). Upon cisplatin treatment, TGCT microtumors also stopped to grow and showed a tumor shrinkage of ~ 55% (Fig. 1D,E). Treatment with both animacroxam and cisplatin was well tolerated, as the survival and embryonic development of the chicken eggs was not affected. At the end of the treatment period, the TGCT tumors were excised from the CAM and histological examination of the proliferation marker Ki67 was performed showing the reduced proliferative activity of animacroxam‐treated tumor cells (Fig. 1F,G).

### 3.2 Animacroxam induces necrotic ‘cap formation’ and reduces glucose uptake

**Figure 2:**
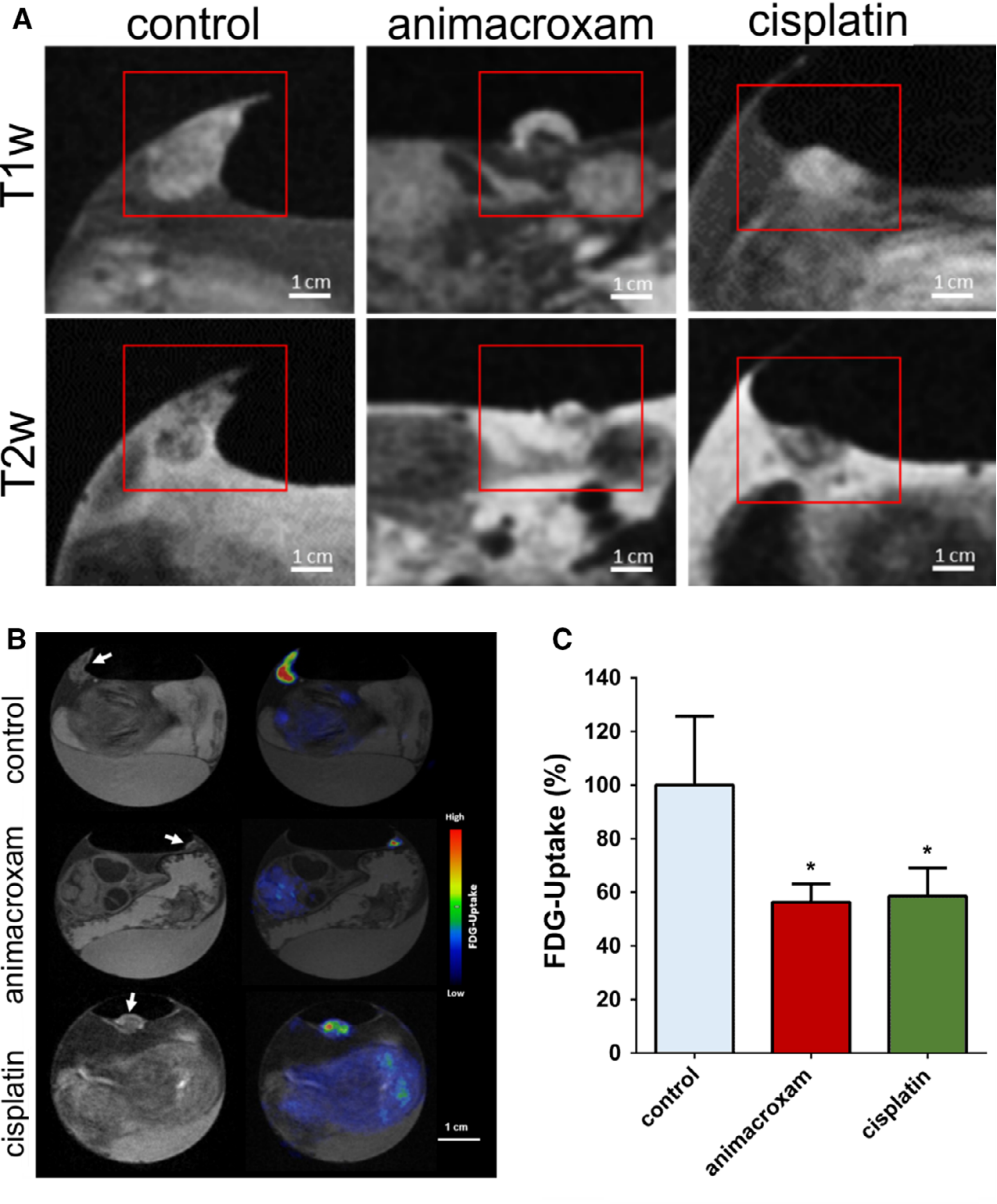
Animacroxam induces ‘cap formation’ and suppresses glucose uptake in inoculated TGCT plaques. (A) T1w and T2w images of control‐, animacroxam‐, and cisplatin‐treated tumor plaques. Animacroxam‐treated tumors show a distinct viable tumor core (dark in T1w‐/bright in T2w images) and a necrotic cap (bright in T1w‐/dark in T2w images). (B) T1w and corresponding PET‐MR images of 18F‐FDG uptake in tumor plaques treated with NaCl, animacroxam, or cisplatin. (C) Animacroxam (5.0 µm)‐ and cisplatin (2.5 µm)‐treated tumor plaques showed a reduced uptake of 18F‐FDG. Results are shown as means ± SEM of at least *n* = 3 independent experiments. Scale bar = 1 cm. *P‐values of ≤ 0.05, unpaired *t*‐test.

Magnetic resonance imaging analysis of animacroxam‐treated tumors revealed an additional morphological finding that can be best described as ‘cap formation of necrotic tumor tissue/debris on top of the tumor’ (bright signal in T1w images, dark in T2w images), a feature that did not occur in cisplatin‐ or vehicle‐treated tumors (Fig. 2A).

To decipher animacroxam’s antineoplastic mode of action, we tested for changes in the energy metabolism and investigated the glucose uptake of the tumors by 18F‐FDG‐PET (Fig. 2B). Compared to control tumors, animacroxam‐ and cisplatin‐treated tumors showed a significant reduction of 18F‐FDG uptake of 43.7% (animacroxam) and 41.6% (cisplatin), respectively (Fig. 2C). Interestingly, the ‘necrotic cap’ seen in the animacroxam‐treated tumor plaques did not show any 18F‐FDG signal, supporting the idea that the tumor capping of animacroxam‐treated tumors reflects an accumulation of protein‐rich fluid and cellular debris of necrotic cells and does not consist of metabolically active tumor cells.

**Figure 3:**
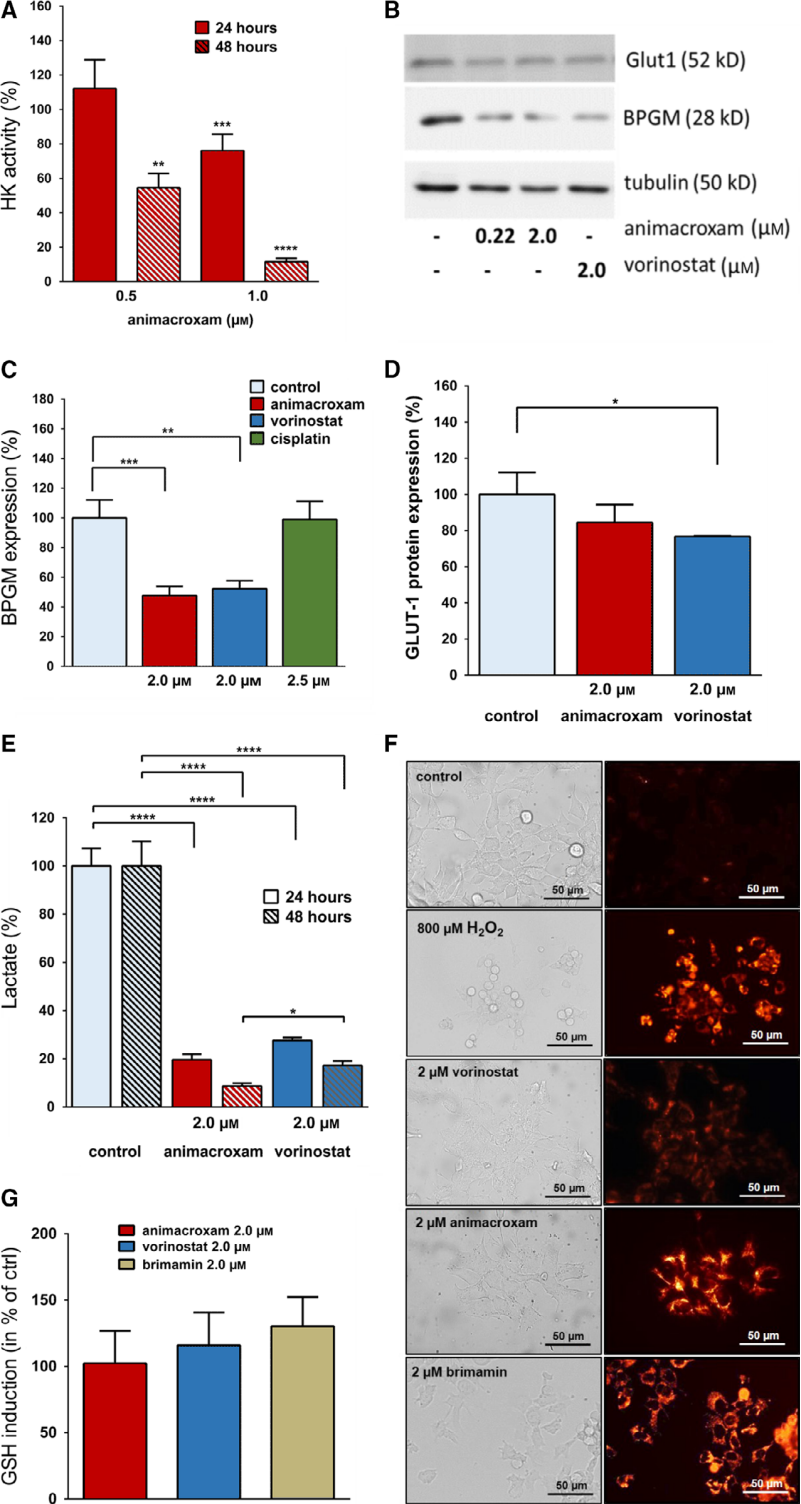
Animacroxam suppresses glycolytic activity of TGCT cells and is associated with elevated levels of ROS. (A) Time‐ and dose‐dependent reduction of HK enzyme activity in TGCT cells following incubation with animacroxam (0.5–1.0 μm) for 24 and 48 h. (B) Western blot analysis shows expression of BPGM and GLUT‐1 in TGCT cells after treatment with animacroxam or vorinostat (2.0 μm). (C) Protein quantification of BPGM expression after incubation with animacroxam, vorinostat, and cisplatin. (D) Protein quantification of GLUT‐1 expression after incubation with animacroxam and vorinostat. (E) Measurement of lactate levels in TGCT cells shows that animacroxam and vorinostat lead to a pronounced and time‐dependent repression of glycolysis after 24 and 48 h, respectively. (F) Detection of ROS in TGCT cells. Left; bright‐field images of untreated, H_2_O_2_ (800 µm), vorinostat (2.0 μm)‐, animacroxam (2.0 μm)‐, and brimamin (2.0 µm)‐treated cells. The right panel shows cellular CellROX Orange fluorescence indicating cytoplasmic ROS within the cells. H_2_O_2_ served as a positive control; scale bar = 50µm. (G) Quantification of the glutathione (GSH) production after incubation with animacroxam (2.0 µm), vorinostat (2.0 µm), and brimamin (2.0 µm). Results are shown as means ± SEM of at least *n* = 3 independent experiments. *P‐values of ≤ 0.05, **P‐values of ≤ 0.005, ***P‐values of ≤ 0.0005, ****P‐values of ≤ 0.0001; one‐way ANOVA *post hoc* Tukey’s test.

To strengthen the idea of an inhibited glucose metabolism or glycolytic flux, respectively, we further examined animacroxam treatment‐induced changes in HK activity *in vitro.* HKs are key enzymes of cellular glucose metabolism, phosphorylating glucose that has been taken up by GLUTs to glucose‐6‐phosphate (G‐6‐P). The conversion to G‐6‐P is an essential step to prevent the back‐diffusion of glucose from the cell to the blood. Moreover, the formation of G‐6‐P enables glucose to enter glycolysis. Treatment with animacroxam led to a significantly decreased HK activity in TGCT tumor cells in a concentration‐ and time‐dependent manner leading to reduction of HK activity of > 85% after 48 h (Fig. 3A). Concomitantly, the expression of the glycolytic enzyme 2,3‐BPGM, which has been suggested to serve as a crucial factor for serine biosynthetic fluxes and thus being of particular importance for proliferation and migration of cancer cells (Oslund *et al.*, 2017), showed a significant drop of about 50% after incubation with animacroxam or vorinostat (Fig. 3B,C).

Interestingly, BPGM reduction occurred only after treatment with the two HDACi compounds animacroxam and vorinostat, but not after treatment with the DNA‐damaging agent cisplatin (Fig. 3C).

A reduced glucose uptake capacity can rely on changes in the expression or activity of glucose transporters. Therefore, we tested for the suppression in GLUT1 expression, as GLUT1 is the most prominent expressed glucose transporter of 2102EP cells (Younes *et al.*, 1996). However, compared to vorinostat, no significant suppression in GLUT1 protein expression (Fig. 3B,D) was observed after 24 h of treatment with animacroxam. As GLUT1 expression showed no significant inhibition by animacroxam, we checked whether animacroxam might rather affect intracellular glucose utilization to impair glycolysis by substrate limitation. Hence, we determined changes in the glycolytic flux by measuring lactate levels in the supernatant of 2102EP cells after incubation with animacroxam and vorinostat which showed that the high glycolytic activity of untreated TGCT cells was dose‐dependently inhibited by animacroxam by up to almost 100% (Fig. 3E). Treatment with vorinostat also repressed the glycolytic activity of TGCT cells, albeit to a lesser extent.

Interestingly, we observed that the animacroxam‐induced breakdown in glycolytic activity was accompanied by elevated ROS levels, as the treatment with animacroxam for 24 h resulted in an increase in cytosolic ROS of 2102EP (Fig. 3F). By contrast, vorinostat only marginally induced ROS in TGCT cells. ROS elevation might occur due to an enhanced ROS production or an inhibition of the ROS‐scavenging glutathione (GSH) system. Performing GSH assays showed that neither animacroxam nor vorinostat inhibited the antioxidant system, as in both cases, GSH levels were not altered (Fig. 3G). However, since animacroxam, but not vorinostat, strongly increased cytoplasmic ROS, this ROS increase may not be due to an HDAC‐inhibitory effect but rather be connected to the cytoskeleton‐interfering imidazole moiety of the chimeric compound animacroxam. Performing ROS experiments with brimamin reflecting the imidazole part of the chimeric inhibitor animacroxam revealed that brimamin induced a pronounced increase in cytoplasmic ROS of TGCT cells (Fig. 3F).

### 3.3 Antiangiogenic effects of animacroxam *in vivo*

**Figure 4:**
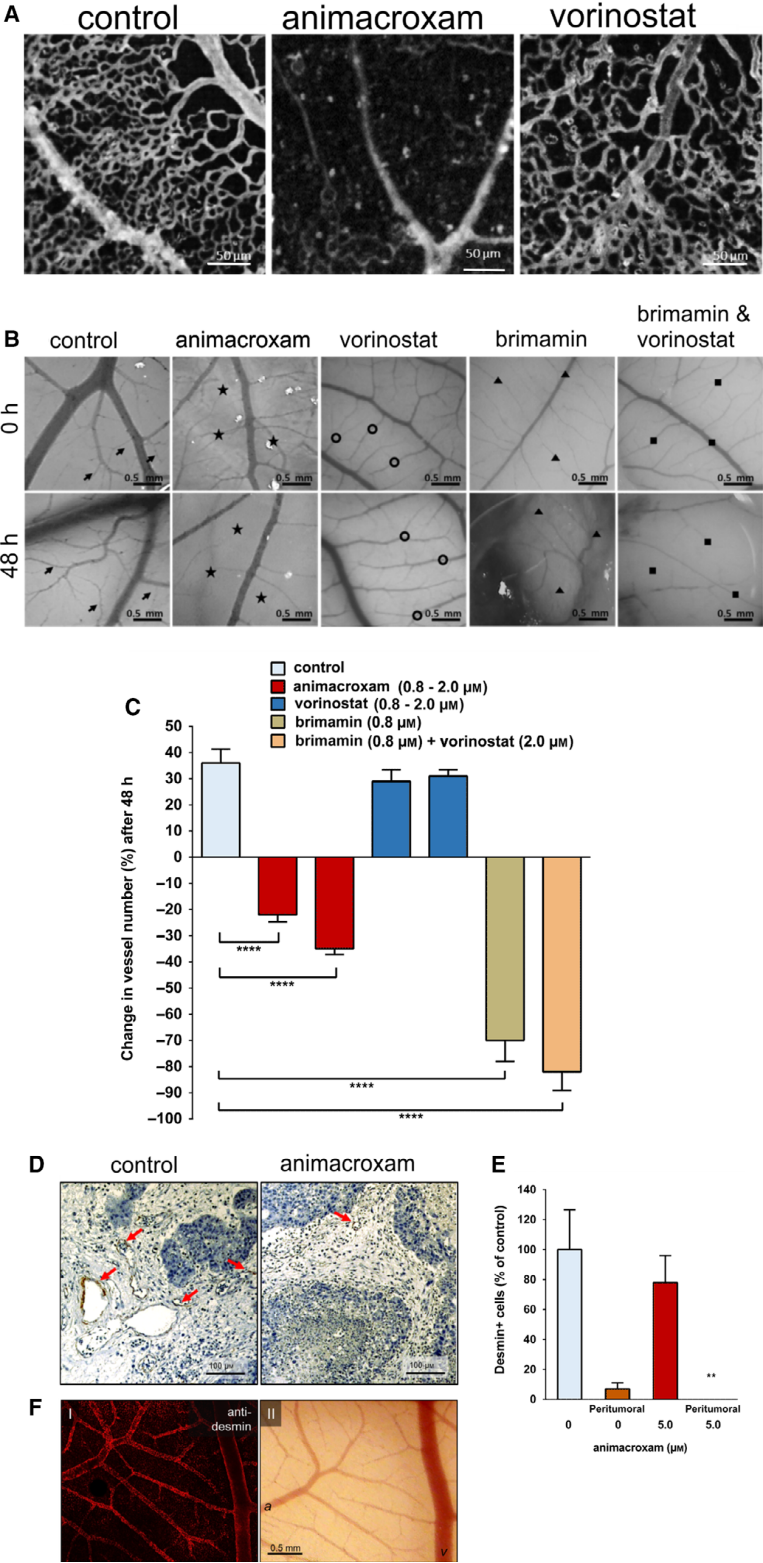
Antiangiogenic and avascular effects of animacroxam and vorinostat on the CAM. (A) Representative images of the microvascular perfusion of the CAM capillary bed. Perfusion is reduced after 48 h of incubation with animacroxam (0.8 µm), but not after incubation with vorinostat (0.8 µm), when compared to PBS‐treated control; scale bar = 50 µm. (B) Images showing macrovascular network of the CAM before and after 48 h of treatment animacroxam (2.0 µm), vorinostat (2.0 µm), brimamin (0.8 µm), or brimamin (0.8 µm) combined with vorinostat (2.0 µm). Arrows indicate sprouting of vessels, stars show vessel regression, triangles show vessel disturbance, scale bar = 0.5mm. (C) Quantification of blood vessel number. (D) Desmin staining (brown) of CAM tumor plaques showed a reduction in vessel formation after animacroxam treatment. (E) Quantification of desmin‐positive cells from intra‐ and peritumoral areas of CAM tumors; scale bar = 100µm. (F) Confocal imaging of CAM blood vessels stained for desmin; scale bar = 0.5mm. (I) Desmin fluorescence is intense in the vessel wall of arteries and veins. (II) Intravital microscopy of the same CAM vascular bed used for staining* a* = arteries *v* = veins. Results are shown as means ± SEM of at least *n* = 3 independent preparations. *P‐values of ≤ 0.05, **P‐values of ≤ 0.005, ***P‐values of ≤ 0.0005, ****P‐values of ≤ 0.0001; one‐way ANOVA *post hoc* Tukey’s test in Fig. 4C and unpaired *t*‐test in Fig. 4E.

A reason for the tumor reductive activity of animacroxam *in vivo* may also be attributed to inhibition of angiogenesis. Changes in capillary plexus perfusion of the CAM became apparent when performing intravital microscopy. As the CAM is a fast‐developing structure, topical treatment with animacroxam for 48 h led to a marked reduction in microvascular perfusion of the treated area as compared to an untreated control area of the same CAM (Fig. 4A). This shows that topical treatment rapidly induces changes on the capillary plexus. Interestingly, neither a reduction in microvessel density nor a reduced perfusion was observed after application of vorinostat, implicating that HDAC inhibition alone may not be enough to induce antiangiogenic effects.

To further characterize the antiangiogenic effects of animacroxam, the number of newly developed larger blood vessels in treated and untreated areas of the CAM was evaluated and quantified by stereo microscopy (Fig. 4B,C). Under control conditions, the vessel density increased by 36% within 48 h, indicating sustained angiogenesis within the developing CAM. Compared to that, the number of blood vessels in animacroxam‐treated CAM areas was significantly decreased. 48 h of animacroxam treatment not only inhibited angiogenic blood vessel formation but even significantly decreased the number of vessels below the initial vessel number at the beginning of the treatment by 35%, which is an indicator for distinct avascular effects (Fig. 4C). Interestingly, vorinostat treatment did not inhibit angiogenesis but rather showed a control like angiogenic growth pattern with increases in the number of blood vessels of up to 31% after 48 h. To decipher whether the imidazole moiety or the HDAC‐inhibitory part of animacroxam may be responsible for the antiangiogenic/avascular effects of animacroxam, additional experiments were performed with the cytoskeleton‐interfering compound brimamin, which is the parent compound of the imidazole‐pharmacophore of animacroxam. Brimamin reduced the number of vessels by up to 70% after 48 h. The combination of brimamin with vorinostat did not lead to synergistic effects (Fig. 4C). The antiangiogenic/avascular effects of animacroxam may therefore be rather attributed to the cytoskeleton‐interfering effects of the 4,5‐diarylimidazole moiety of the compound than to its HDAC‐inhibitory portion, as HDAC inhibition alone was not able to inhibit angiogenesis.

Changes in the microvessel density of tumor plaques excised from the CAM were determined by desmin staining, as desmin is expressed by arterial as well as venous blood vessels of the CAM vascular network (Fig. 4F) and is known to serve as a specific marker for CAM micovessels (Nitzsche *et al.*, 2012). For quantification, desmin‐positive cells were counted in 10 randomly chosen HPF per sample section, which revealed a 22% reduction in microvessel density of animacroxam‐treated tumors. Moreover, peritumoral vessel expression was significantly abolished in animacroxam‐treated tumors (Fig. 4D,E).

### 3.4 Animacroxam inhibits endothelial tube formation and endothelial gap‐junctional communication

**Figure 5:**
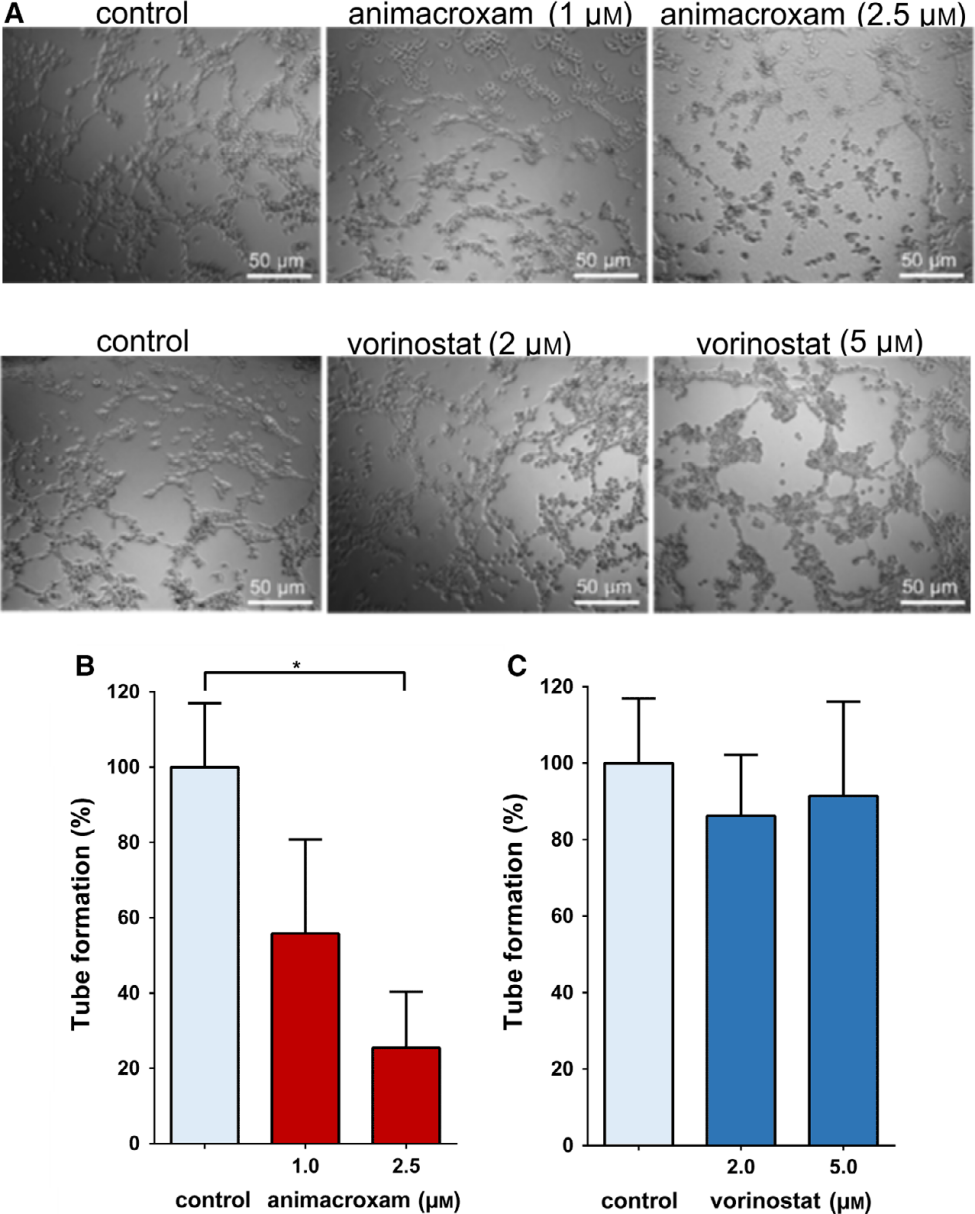
Capillary‐like tube formation is reduced after incubation with animacroxam. (A) Representative images of untreated and animacroxam (1.0–2.5 µm) EA.hy926 cells show a strong and dose‐dependent reduction of tube‐like structures after animacroxam treatment when compared to the control (100%). EA.hy926 cell‐treated vorinostat (2.0–5.0 µm) displayed only minor reductions of tube‐like structures when compared to the control (100%); scale bar = 50 µm. (B) Quantification of the tube formation compared to the untreated control after incubation with animacroxam (1.0–2.5 µm). (C) Quantification of the tube formation compared to the untreated control after incubation with vorinostat (2.0–5.0 µm). Results are shown as means ± SEM of *n* = 3 independent experiments. *P‐values of ≤ 0.05; one‐way ANOVA *post hoc* Tukey’s test.

Differences in the antiangiogenic potency of animacroxam and vorinostat were further reflected in tube formation assays modeling angiogenic reorganization *in vitro* (Fig. 5A). Treatment of endothelial EA.hy926 cells with animacroxam resulted in a significant and dose‐dependent reduction in the formation of capillary‐like tube structures of up to 73.2% after 16 h (Fig. 5B). Vorinostat was not able to inhibit tube formation significantly; even at a high vorinostat concentration of 5.0 µm, only marginal reduction of 13.4% was observed (Fig. 5C).

**Figure 6:**
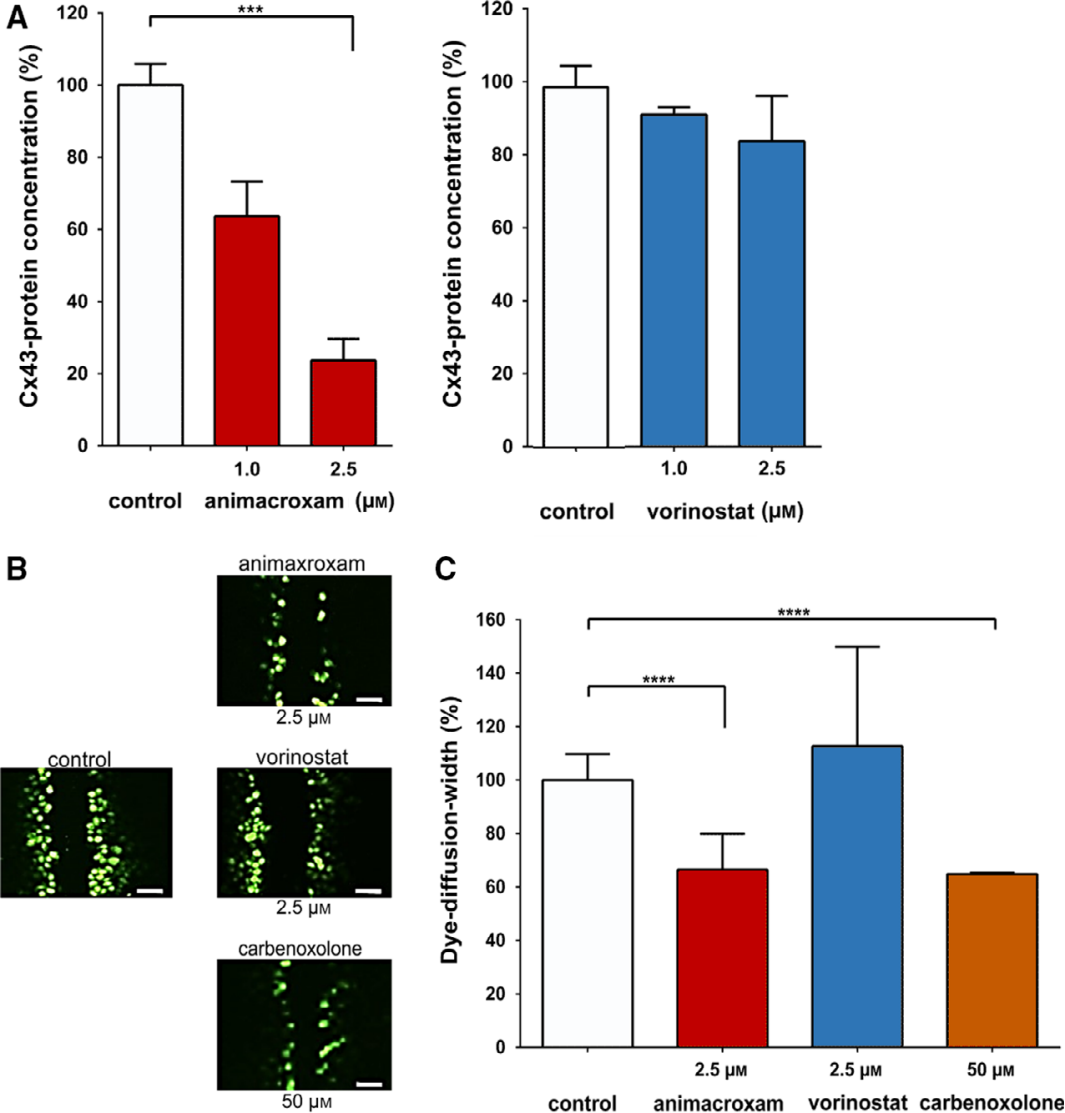
Treatment‐induced changes in Cx43 expression and endothelial cell–cell communication. (A) Protein expression of Cx43 was reduced after 24 h of incubation with animacroxam (1.0–2.5 µm) or—albeit to a lesser extent—with vorinostat (1.0–2.5 µm) when compared to control (100%). (B) Representative images of Lucifer Yellow dye diffusion in endothelial EA.hy926 cells after incubation with animacroxam or vorinostat; scale bar = 100 µm. (C) Lucifer Yellow dye diffusion, indicating intercellular gap‐junctional cell–cell coupling, was diminished after 24 h of incubation with animacroxam (2.5 µm). This effect was absent after incubation with vorinostat (2.5 µm). The established Cx43 inhibitor Cbx (50 µm) served as a positive control. Results are shown as means ± SEM of at least *n* = 3 independent experiments. ***P‐values of ≤ 0.0005, ****P‐values of ≤ 0.0001; one‐way ANOVA *post hoc* Tukey’s test.

The underlying molecular events of the antiangiogenic/avascular effects were further investigated by checking the impact of animacroxam on endothelial cell–cell communication via gap junctions. Gap‐junctional communication of microvascular endothelial cells is a prerequisite for the adjacent vascular adaptation and angiogenic growth of capillary networks (Pries *et al.*, 2010). As the changes in cell–cell communication occur prior to changes of the capillary perfusion or development of the CAM, time‐points of 24 h were chosen for the following *in vitro* investigations. Gap junctions are built of connexins that form intercellular communication pores allowing signaling molecules to diffuse rapidly and directly between endothelial cells (Connors, 2012). With respect to the information transfer capability of the gap junction‐forming connexin 43 (Cx43), it has recently been shown to be of particular importance for endothelial cell–cell communication (Pogoda *et al.*, 2019). Thus, we tested whether Cx43‐mediated gap‐junctional communication may account for the impaired capillary blood perfusion, reduced angiogenesis and avascular effects of animacroxam (Fig. 6). While in animacroxam‐treated endothelial cells, the expression of Cx43 protein dropped significantly by up to 76.4 ± 6%, vorinostat‐induced inhibition of Cx43 protein led to a nonsignificant reduction of 16.3 ± 12% only (Fig. 6A).

To verify the impact of gap‐junctional Cx43 protein expression on endothelial cell–cell communication at functional level, we performed scrape loading assays in which the gap junction‐specific diffusion of the highly fluorescent dye Lucifer Yellow is determined in living cells (Bader *et al.*, 2017) (Fig. 6B). Compared to untreated cells, animacroxam‐treated EA.hy926 cells showed a decrease in the intercellular diffusion of Lucifer Yellow of up to one‐third (33.5 ± 6.7%) after 24 h (Fig. 6B,C). The effect of animacroxam was in the range of that of Cbx, a well‐known gap junction/Cx43 uncoupling agent, which served as a positive control (Sagar and Larson, 2006). Noteworthy, vorinostat failed to decrease gap‐junctional dye diffusion when given at an equimolar concentration of 2.5 µm (Fig. 6B,C), further supporting the interpretation that HDAC inhibition alone may not be sufficient to decrease gap‐junctional Cx43 expression and related cell–cell communication.

## 4 Discussion

In the present study, the antineoplastic and antiangiogenic effects of the novel chimeric HDAC inhibitor animacroxam were investigated in xenografted models of testicular germ cell tumors (TGCTs) and the underlying modes of action were deciphered. TGCT was used exemplarily for a broad range of human cancers, which have been shown to respond to animacroxam *in vitro*. In previous studies, the *in vitro* effectiveness of animacroxam in TGCT cells was shown to be as pronounced as cisplatin which reflects the standard chemotherapeutic for medical TGCT treatment (Mahal, Schruefer, *et al.*, 2015; Steinemann *et al.*, 2017). Moreover, animacroxam also proved its suitability for the treatment of cisplatin‐refractory/insensitive cancer cells as its modes of action do not require the formation of DNA intrastrand crosslink adducts, but rather attacks cancer cells at the level of cytoskeletal integrity and HDAC activity.

Therapies beyond the standard therapeutics for cancer and especially those targeting epigenetic regulators such as HDACs have gained increasing attention during the past decades. In the course of this development, the inhibition of HDACs has already been evaluated for solid tumors in some preclinical studies and early clinical trials (Richon *et al.*, 2009; Rompicharla *et al.*, 2018; Siegel *et al.*, 2009). Generally, HDAC‐inhibiting approaches are well tolerated (Pili *et al.*, 2012; Siu *et al.*, 2008) and show promising results in TGCT (Münster *et al.*, 2007). Hence, the investigations point toward a potential of HDAC inhibition as a therapeutic option, but also show that the spectrum of effectiveness strongly depends on the specific mode of HDAC inhibition of the different classes of inhibitors (short‐chain fatty acids, benzamides, cyclic tetrapeptides, sirtuin inhibitors, and hydroxamic acids) that have been investigated (Eckschlager *et al.*, 2017).

In our study, the chimeric inhibitor animacroxam, bearing a hydroxamic acid moiety for HDAC inhibition, exerted antineoplastic potency in TGCTs grown in the flank of athymic nude mice. The treatment was well tolerated since food and water consumption of the mice remained unchanged, and no treatment‐induced weight loss or changes in the animals’ behavior was observed. The data confirmed our previous results on low toxicity (Steinemann *et al.*, 2017) and high tolerability (Höpfner *et al.*, 2018) of the novel hybrid compound. Based on the initial mouse experiments, additional *in vivo* studies were performed employing an advanced CAM assay in which the growth of TGCT plaques and their metabolic activity was assessed by MR/PET imaging, a technique that is well established for the clinical assessment of tumor development and metabolic tumor activity in patients (Gebhardt *et al.*, 2013; Zuo *et al.*, 2015).

Chorioallantoic membrane assays confirmed the preliminary mouse data displaying an increase in tumor volume of untreated control tumors of more than 114% after 7 days, while animacroxam treatment not only slowed down the tumor growth but even caused a shrinkage of the tumors by more than 60% below the initial tumor volume at the beginning of the experiments. Although immunohistological (IHC) staining with Ki67 (Rose *et al.*, 1996) points toward a correlation between the observed tumor reduction and a decreased cellular proliferation rate of animacroxam‐treated tumors, further investigations on bigger sample sizes are warranted to carve out statistical significance.

Applying MRI‐based morphological evaluations of tumor‐bearing CAMs revealed interesting morphological changes in animacroxam‐treated tumors. We observed necrotic caps of protein‐rich fluid and necrotic debris sitting on top of animacroxam‐treated tumors. This cap formation was only seen in animacroxam but not in cisplatin‐treated tumors. Interestingly, such protein‐rich edema has been described in inflammatory diseases where endothelia lose their junctional integrity resulting in leakiness of the endothelial barrier (Di *et al.*, 2016). Our data on changes in endothelial permeability have been associated with elevated levels of ROS that represent the primary cause of endothelial dysfunction (Incalza *et al.*, 2018). Interestingly, we observed increased ROS formation in tumors treated with animacroxam. Hence, we propose that animacroxam may also influence endothelial permeability, which has not been observed for cisplatin or the HDACi vorinostat, respectively.

As tumors often display an aberrant glucose energy metabolism relying on the predominant production of ATP by glycolysis (Warburg effect), we elucidated the effects of animacroxam on enzymes involved in glucose uptake and glycolysis. It has been shown for several human tumors that the high‐glucose utilization of tumor cells is correlated with an overexpression of glucose transporters and HKs (Binderup *et al.*, 2013). The glucose transporter Glut‐1, which is expressed in 2102EP‐derived TGCT, was not significantly inhibited by animacroxam, but the activity of the HK was strongly reduced. Furthermore, we observed that animacroxam caused downregulation of the glycolytic enzyme BPGM. It has recently been shown that 2,3‐BPG, produced by BPGM, exerts functions apart from hemoglobin binding (Carreras *et al.*, 1986). One such biological function of BPGM is to control glycolytic intermediate levels to mediate serine biosynthetic fluxes which are essential for macromolecular biosynthesis for rapid cancer cell growth and proliferation (Oslund *et al.*, 2017). BPGM reduction was seen after treatment with the animacroxam and the HDAC‐inhibiting vorinostat, but not after treatment with the DNA‐damaging agent cisplatin. Thus, it is feasible that BPGM inhibition by animacroxam might be an HDACi‐driven effect. Animacroxam treatment also resulted in an almost complete breakdown in glycolysis, shown by dramatically lowered lactate levels. However, it remains to be elucidated whether this breakdown in glucose metabolism was causative for the antitumor effects or rather occurred as a consequence of animacroxam‐induced cell death.

*In vivo* PET analysis using 18F‐FDG, a surrogate tracer for glucose, revealed a significant reduction of the glucose metabolism in TGCT of more than 40% after treatment with animacroxam. The pronounced uptake of intravenous applied 18F‐FDG of the control tumors affirmed the connection of the vascular system of the CAM to the TGCTs via tumor angiogenesis. In histological sections of paraffin‐embedded control tumors, a successful sprouting of vessels into the inoculated TGCT microtumors was detected by desmin‐positive staining of (peri‐)tumoral CAM vessels. Animacroxam treatment diminished the number of vessels and especially peritumoral vessel expression was significantly inhibited, pointing toward an antiangiogenic effect of animacroxam *in vivo*.

Histone deacetylase inhibitors have previously been shown to inhibit angiogenesis via suppression of proangiogenic genes (Roberto Pili *et al.*, 2017; Qian *et al.*, 2004) or the ERK1/2‐MAP kinase pathway (Duan *et al.*, 2017). HDAC inhibition by imidazole derivatives was also reported to result in a reduced formation of new blood vessels (Takahashi *et al.*, 1998). Bonezzi et.al. described that the cytoskeleton integrity of human endothelial cells (HUVEC) is disrupted after treatment with aptly substituted 4,5‐diarylimidazoles, which is in line with the results of our previous study showing vascular disruptive properties of imidazole‐based animacroxam (Bonezzi *et al.*, 2009). This might be of particular interest as antiangiogenic properties of novel anticancer agents not only comprise the inhibition of tumor vessel sprouting but also the induction of a collapse of the existing tumor vasculature by impairing vascular remodeling. Here, we show that the multimodal HDACi animacroxam not only reduces tumor angiogenesis but partially also acts as an avascular compound. The mechanism by which animacroxam exerts its effects on endothelial vessel formation and maintenance may be explained by its HDAC‐inhibitory potency, including a proposed specificity for the cytoplasmic HDAC6 (Mahal, Schruefer, *et al.*, 2015). The latter is known to be involved in antiangiogenic and avascular signaling by modulating cytoskeleton and protein trafficking dynamics. Determination of the expression pattern of HDAC class I‐IV family members in the tumor (2102EP) and endothelial cells (Ea.hy926) used in this study revealed that the class I family members HDAC1, HDAC2, and HDAC3 are the primarily expressed HDACs in both cell lines (Fig. S1A). In accordance with our findings, *Fritzsche et al.* also showed that HDAC‐1, HDAC‐2, and HDAC‐3 are upregulated in TGCT tissue samples (Fritzsche *et al.*, 2011). Interestingly, the expression of HDAC‐1, HDAC‐2, and HDAC‐3 in 21012EP cells was downregulated upon animacroxam or vorinostat treatment (Fig. S1B). Thus, in forthcoming studies it will be interesting to examine the importance of the downregulation in the expression of these members of the HDAC family for the antiproliferative effects of animacroxam in TGCTs.

Avascular effects of animacroxam may also be related to the additional involvement of the cytoskeleton‐interfering imidazole moiety which has already shown to disrupt blood vessels (Mahal, Biersack, *et al.*, 2015). An indication for the involvement of the cytoskeleton‐interfering imidazole moiety on the effects of animacroxam is coming from findings on brimamin, resembling the avascular disruptive imidazole moiety of animacroxam. In CAM experiments, brimamin exerted pronounced antiangiogenic/avascular effects while vorinostat, resembling the HDAC‐inhibitory moiety of animacroxam, failed to induce antiangiogenic or even avascular effects. Moreover, only animacroxam was able to induce a pronounced inhibition of capillary‐like tube formation of EA.hy926 cells, while vorinostat failed to induce antiangiogenic effects.

A functional cytoskeleton plays a key role in the stability and migration of blood vessel‐forming endothelial cells (Steinemann *et al.*, 2017). Reorganization of the cytoskeletal filaments by animacroxam may also cause the disruption of cell differentiation and vessel maturation thereby leading to antiangiogenic effects (Kanthou and Tozer, 2007, 2009). The antiangiogenic properties of animacroxam may be of great importance especially for the treatment of highly vascularizing tumors. Angiogenesis is also required for metastatic dissemination of cancer cells (Silván *et al.*, 2015). Therefore, the effects of animacroxam were evaluated on the capillary level where a strongly reduced angiogenesis as well as a markedly reduced blood perfusion was found in the treated microvascular areas. This is in line with the reduced vascularization found in the histological and morphological CAM analysis.

The expression of the most important vascular endothelial connexin, Cx43, which is essential for gap‐junctional intercellular communication (GJIC) (Pogoda *et al.*, 2019), was significantly inhibited by animacroxam. Our findings are in line with a study from *Sato et al.* who observed that a reduction in GJIC communication activity is correlated with a reduction in Cx43 protein levels (Sato *et al.*, 2002). By contrast, the HDACi vorinostat was not able to inhibit angiogenesis and did not induce avascular areas on the CAM nor affected Cx43 expression. Our data implicate that the cytoskeleton reorganizing properties of animacroxam may additionally be required to induce full‐range antiangiogenic and avascular effects of the compound and will be investigated in depth in a forthcoming study.

## 5 Conclusions

In summary, we show that the novel chimeric inhibitor animacroxam potently reduces tumor growth and angiogenesis and may thus be an interesting alternative for medical therapy of cancers. As animacroxam was as effective as the standard chemotherapeutic cisplatin, animacroxam may be of particular interest when patients cannot be treated with platinum‐based chemotherapy or are refractory to cisplatin treatment. Compared to pan‐HDACi such as vorinostat, the conjugation of a pan‐HDAC‐inhibitory hydroxamic acid residue with a cytoskeleton‐interfering imidazole‐based pharmacophore was shown to exert superior effectiveness. Thus, our results make animacroxam a promising candidate for the development of novel approaches beyond standard platinum‐ or HDACi‐based chemotherapy.

## Conflict of interest

The authors declare no conflict of interest.

## Author contributions

GS, BN, and MH conceived and designed the research. GS, AD, JS, DJ, MF, NB, EJK, and TM performed the experiments. GS, AD, JS, DJ, MF, NB, EJK, TM, BN, and MH analyzed the data. GS, AD, JS, DJ, MF, BB, WB, BN, and MH interpreted results of experiments. GS, AD, JS, MF, BN, and MH prepared figures. GS, MF, BB, TM, BN, and MH drafted, edited, and revised the manuscript. GS, AD, JS, DJ, MF, BB, NB, EJK, RS, WB, TM, BN, and MH approved the final version of the manuscript.

## Supporting information


**Fig S1.** Relative expression of HDAC subtypes in TGCT cells (2120EP) and endothelial cells (EA.hy926). (A) Pie charts showing the relative amount of HDAC RNA in % measured in TGCT and endothelial cells by real‐time‐PCR (RT‐PCR), in relation to overall HDAC expression. Class I HDACs (HDAC1, HDAC2, and HDAC3) are primarily expressed in both cell types. (B) Changes in mRNA expression of HDAC subtypes in TGCT cells after treatment with animacroxam or vorinostat for 24 h compared to untreated cells.
