# Supplementary figures and images for: Antitumor and antiangiogenic activity of the novel chimeric inhibitor animacroxam in testicular germ cell cancer
Source: Mol Oncol. 2019 Oct 22;13(12):2679–96. doi: 10.1002/1878-0261.12582 (PMC6887589; doi:10.1002/1878-0261.12582)

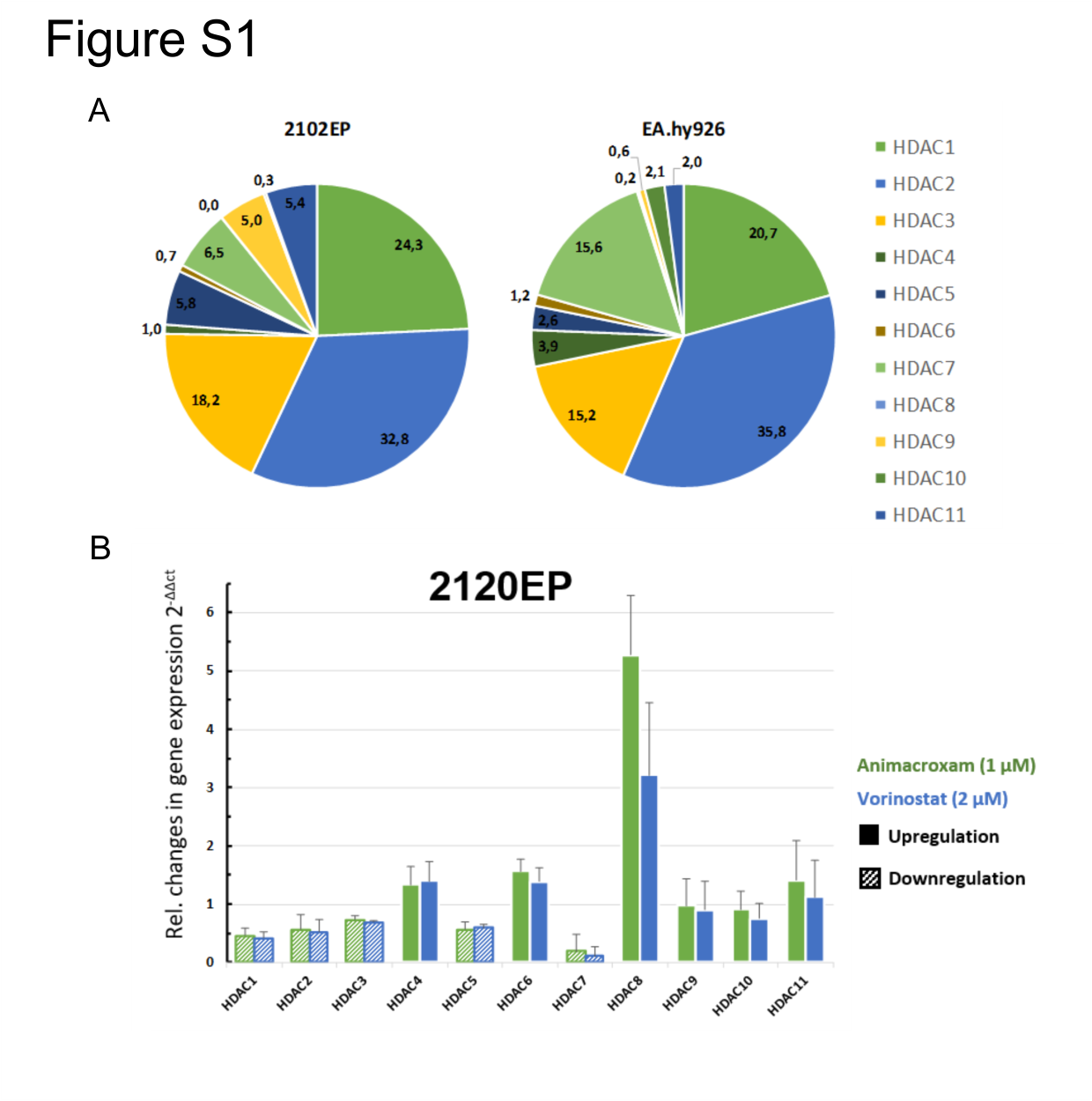

Supplement: Supplementary file 1 — Fig S1. Relative expression of HDAC subtypes in TGCT cells (2120EP) and endothelial cells (EA.hy926). (A) Pie charts showing the relative amount of HDAC RNA in % measured in TGCT and endothelial cells by real‐time‐PCR (RT‐PCR), in relation to overall HDAC expression. Class I HDACs (HDAC1, HDAC2, and HDAC3) are primarily expressed in both cell types. (B) Changes in mRNA expression of HDAC subtypes in TGCT cells after treatment with animacroxam or vorinostat for 24 h compared to untreated cells. [file MOL2-13-2679-s001.tif]
